# Major Dietary Patterns in Relation to General and Central Obesity among Chinese Adults

**DOI:** 10.3390/nu7075253

**Published:** 2015-07-15

**Authors:** Canqing Yu, Zumin Shi, Jun Lv, Huaidong Du, Lu Qi, Yu Guo, Zheng Bian, Liang Chang, Xuefeng Tang, Qilian Jiang, Huaiyi Mu, Dongxia Pan, Junshi Chen, Zhengming Chen, Liming Li

**Affiliations:** 1 Department of Epidemiology and Biostatistics, School of Public Health, Peking University Health Science Center, 38 Xueyuan Road, Beijing 100191, China; E-Mails: yucanqing@pku.edu.cn (C.Y.); lvjun@bjmu.edu.cn (J.L.); 2 Discipline of Medicine, University of Adelaide, SAHMRI, North Terrace, Adelaide, South Australia 5000, Australia; E-Mail: zumin.shi@adelaide.edu.au; 3 Clinical Trial Service Unit and Epidemiological Studies Unit (CTSU), Nuffield Department of Population Health, University of Oxford, Richard Doll Building, Old Road Campus, Oxford OX3-7LF, UK; E-Mails: huaidong.du@ctsu.ox.ac.uk (H.D.); zhengming.chen@ctsu.ox.ac.uk (Z.C.); 4 Department of Nutrition, Harvard School of Public Health, 665 Huntington Ave, Boston, MA 02115, USA; E-Mail: nhlqi@channing.harvard.edu; 5 Channing Division of Network Medicine, Department of Medicine, Brigham and Women’s Hospital and Harvard Medical School, 75 Francis Street, Boston, MA 02115, USA; 6 Chinese Academy of Medical Sciences, Fuwai Hospital Xishan Branch Court, Western Feng Cun, Mentougou, Beijing 102308, China; E-Mails: guoyu@kscdc.net (Y.G.); bianzheng@kscdc.net (Z.B.); 7 Henan Center for Disease Control and Prevention, 105 Nongye East Road, Zhengzhou 450016, China; E-Mail: hnchangliang@sina.com; 8 Sichuan Center for Disease Control and Prevention, 6 Zhongxue Road, Chengdu 610041, China; E-Mail: sccdctxf@163.com; 9 Department of Non-communicable Diseases, Liuzhou Center for Disease Control and Prevention, 1-1 Tanzhong West Road, Liuzhou 545007, China; E-Mail: lzjiangqn@163.com; 10 Department of Non-communicable Diseases, Nangang Center for Disease Control and Prevention, 225 Wenchang Street, Haerbin 150040, China; E-Mail: ztmhy@126.com; 11 Department of Non-communicable Diseases, Tongxiang Center for Disease Control and Prevention, 64 Maodun East, Wutong Town, Tongxiang 314500, China; E-Mail: dongxia0724_pan@163.com; 12 China National Center for Food Safety Risk Assessment, 37 Guangqu Road, Beijing 100738, China; E-Mail: chenjunshi@cfsa.net.cn

**Keywords:** dietary pattern, general obesity, central obesity, body mass index, waist circumference, cross-sectional study

## Abstract

Limited evidence exists for the association between diet pattern and obesity phenotypes among Chinese adults. In the present study, we analyzed the cross-sectional data from 474,192 adults aged 30–79 years from the China Kadoorie Biobank baseline survey. Food consumption was collected by an interviewer-administered questionnaire. Three dietary patterns were extracted by factor analysis combined with cluster analysis. After being adjusted for potential confounders, individuals following a traditional southern dietary pattern had the lowest body mass index (BMI) and waist circumference (WC); the Western/new affluence dietary pattern had the highest BMI; and the traditional northern dietary pattern had the highest WC. Compared to the traditional southern dietary pattern in multivariable adjusted logistic models, individuals following a Western/new affluence dietary pattern had a significantly increased risk of general obesity (prevalence ratio (PR): 1.06, 95% confidence interval (CI): 1.03–1.08) and central obesity (PR: 1.07, 95% CI: 1.06–1.08). The corresponding risks for the traditional northern dietary pattern were 1.05 (1.02–1.09) and 1.17 (1.25–1.18), respectively. In addition, the associations were modified by lifestyle behaviors, and the combined effects with alcohol drinking, tobacco smoking, and physical activity were analyzed. Further prospective studies are needed to elucidate the diet-obesity relationships.

## 1. Introduction

General obesity, defined by body mass index (BMI), is associated with multiple comorbidities, including cardiovascular disease, diabetes and cancer, and a higher risk of all-cause mortality [1]. Central obesity, defined by waist circumference (WC), is an independent predictor of morbidity and mortality [2]. The cause of obesity is multifactorial and reflects the balance between dietary intake and energy expenditure, such as physical activity [3].

Dietary patterns characterize how foods and nutrients are consumed in combinations, reflect the effects of overall diet, and are more realistic and predictive in analysis of the relationship of diet with health and disease than individual foods or nutrients [4]. Several studies have reported the association of the major dietary patterns with general and central obesity [5,6,7,8,9], and the dietary patterns vary hugely across different countries, culture, or ethnic groups. In general, dietary patterns with greater intakes of high-fiber cereal, fruit, vegetables, and reduced-fat dairy products were inversely associated with obesity, while the dietary patterns with greater intakes of meat, potatoes, and sweets, were positively associated with obesity measures. However, few data are available from developing countries, especially from China, where people have different food culture from other countries and is experiencing an accelerating nutrition transition due to rapid economic, social and cultural changes [10,11], and where the prevalence of general and central obesity has increased greatly during the past decades [12].

Previous studies conducted among the Chinese population reported that dietary patterns and food factors are associated with the presence of glucose tolerace abnormalities [13], metabolic syndrome [14,15], and cardiovascular disease risk factors such as hypertension, hyperglycemia, triglyceride [15]. As to obesity, this association was observed among Chinese men aged 18–59 years for central obesity [15] and among Chinese young women aged 18–44 years for general obesity and central obesity [16]. In the present study, we aimed to examine the associations of dietary patterns with general and central obesity in a large sample of Chinese adults aged 30–79 years from the China Kadoorie Biobank (CKB). We also investigated the joint effects of dietary patterns and lifestyle factors including alcohol drinking, smoking and physical activity.

## 2. Methods

### 2.1. Study Population

The CKB is an ongoing prospective study that was designed to investigate the relationship between socioeconomic status, lifestyle behavior, and environmental factors and their association with chronic diseases such as ischemic heart disease, stroke and cancer. Detailed study objectives and designs are described elsewhere [17,18]. In brief, 512,891 men and women were recruited at a baseline survey in 2004–2008 from the general population residing in five urban areas (Qingdao, Harbin, Haikou, Suzhou and Liuzhou) and five rural areas (Sichuan, Gansu, Henan, Zhejiang and Hunan). Selection of the survey areas was based on local patterns of disease and exposure to certain risk factors, population stability, quality of death and disease registries, and local commitment and capacity. These 10 geographically defined areas were further divided into two groups, southern and northern areas, along the Qinling Mountains-Huaihe River line. Much difference exists in the natural geography, geology and culture between two groups (Supplemental Figure S1).

In each study area, about 100–150 administrative units (rural villages or urban residential communities) were selected for the study based on local records. Invitation letters and study information leaflets were delivered door-to-door by local community leaders or health workers after extensive publicity campaign. The target population of the study was restricted to permanent residents aged 30 years or more because of their higher disease outcomes and lower population mobility than younger adults. Eligible residents were invited to participate in the baseline survey at local assessment centers which were set up in each administrative unit specifically for the study. A team of about 15 full-time staff with medical qualifications and fieldwork experience in each study area was established for the survey. In the present analysis, we excluded participants with a prior history of coronary heart disease (*n =* 15,472), diabetes (*n =* 16,162), cancer (*n =* 2577), or stroke (*n =* 8884); and those with missing value on BMI (*n =* 2). A total number of 194,276 men and 279,916 women remained.

The project was approved by the ethical committee and research council of the Chinese Centre for Disease Control and Prevention (Beijing, China, 005/2004) and the Oxford Tropical Research Ethics Committee at the University of Oxford (UK, 025-04), and informed written consent was obtained from each participant.

### 2.2. Data Collection

At the baseline survey, a standardized questionnaire was face-to-face administered by trained interviewers using a laptop-based data-entry system. Detailed information on socio-demographic status, medical history, and lifestyle behaviors such as smoking habits, alcohol consumption, diet and physical activity, was obtained from each participant. The questions on the usual type and duration of activities related to work, commuting, household chores and leisure-time exercise during the past 12 months, were used to quantify the amount of daily physical activity (in metabolic equivalent hours per day (MET-hours/day)) [19].

Dietary data covered 12 major food groups in China: rice, wheat, other staples, meat, poultry, fish, eggs, fresh fruit, fresh vegetables, preserved vegetables, soybean and dairy products; each with five frequency levels of habitual consumption (never/rarely, monthly, 1–3 days/week, 4–6 days/week or daily) during the past 12 months. Each food intake was recoded as days/week: 0, 0.5, 2, 5, and 7 respectively. In addition, the frequency and quantity of beverages consumption were also recorded, including four types of tea (green/jasmine tea, oolong tea, black tea or other tea) and five types of alcohol (beer, rice wine, wine, spirit with ≥40% alcohol or spirit with <40% alcohol). Thus, the average consumption (in g/week) was calculated [20].

A repeat questionnaire survey was performed within a year after baseline among 926 participants (mean delay of 5.4 months), good reproducibility of the food questionnaire was shown for most of food groups except for fresh vegetables. This is likely due to seasonal availability of fresh vegetables (Supplemental Table S1).

### 2.3. Dietary Patterns

Dietary patterns from the 12 aforementioned food and nine beverage groups were constructed using factor analysis combined with cluster analysis [21,22]. We first applied factor analysis using a principal component method to identify the major common food factors; then, an orthogonal (varimax) rotation was performed to achieve the structure with independent factor and greater interpretability. The number of factors retained by eigenvalue (>1), scree plot, factor interpretability and the variance explained (5%) by each factor. In the end, we chose the two-factor solution. The pattern loadings (see Supplementary Table S2) showed that the first factor, termed “staple food”, showed a negative high loading on rice, and high loadings for wheat and other staple foods. The second factor has high loadings on various “western” and “newly affluent” foods, such as meat, poultry, fish, eggs, soybean, fresh fruit and dairy products (cut-off point: ≥0.4). Totally, these two factors explained 24.38% of the whole variance of food intake frequency scores.

Subsequently, the factor scores for each factor, calculated by summing the consumption of each food group that was weighted by a factor loading, were used in a cluster analysis. Because we had a large number of participants (*n =* 474,192), a two-step approach was applied. First, we performed hierarchical cluster analysis by randomly selecting 1% of the total cases to help identify the appropriate numbers of clusters and to determine reasonable initial cluster centers for a subsequent K-means cluster analysis. In the end, the K-means cluster analysis identified three distinct clusters. To minimize any effect of the ordering of samples, five random variables were created and used to sort the data file in ascending and descending order. Cluster assignment was robust across the ten runs; 99.19% to 99.68% of the participants were assigned to the same cluster in all runs. Furthermore, we ran analysis of variance to test the validity of the classification, which indicated that the segmentation of the three clusters was quite satisfactory (Supplemental Table S3).

### 2.4. Assessment of Anthropometric Measures

Anthropometric measures were also assessed by a baseline survey by trained technicians according to standard protocols. Participants did not wear shoes during the measurements of height and weight. Standing height was measured to the nearest 0.1 cm using a manufactured instrument. Weight was measured to the nearest 0.1 kg using a TANITA TBF-300GS body composition analyzer (Tanita Corp., Tokyo, Japan). BMI was calculated as weight in kilograms divided by the square of the standing height in meters. WC was measured midway between the iliac crest and the lower rib margin at the end of normal expiration. In the present study, we defined general obesity as BMI ≥ 28 kg/m^2^, and central obesity as WC ≥ 80 cm in women and WC ≥ 85 cm in men [23].

### 2.5. Statistical Analysis

The characteristics were compared between the dietary patterns using the logistic models for categorical variables or a general linear model for the continuous variables to adjust for age and sex. Values are presented as mean ± standard error (SE) or proportion respectively. The crude and multivariable-adjusted means and their 95% confidence intervals (CI) of BMI and WC between different dietary patterns were estimated using a generalized linear model. Considering that odds ratio can overestimate the effect in cross-sectional studies with high-prevalence binary outcomes [24], prevalence ratios (PR) and their 95% CI for were estimated using log-binomial regression [25], adjusting for age (continuous), sex (men, or women), study area (north area or urban area, both yes or no), marital status (married, or unmarried), education level (no formal school, primary school, middle school, high school, or college/university), household income (<10,000, 10,000–19,999, ≥20,000 Yuan RMB/year), alcohol consumption (never drinker, occasional drinker, ex-drinker, or current regular drinker), tobacco smoking (never smoker, occasional smoker, ex-smoker, or current regular smoker), and physical activity level in MET-hours/day (continuous). In order to eliminate the reverse effect of unmeasured conditions, we performed two sensitivity analyses. First, we excluded a total number of 4175 participants who died within two years after baseline enrollment, and compared the associations with the whole sample. Second, we excluded a total number of 6029 participants who tried to reduce weight by dieting or using weight-loss drugs in the past 12 months, and reevaluate the association of dietary patterns with general and central obesity.

We also performed joint analyses to compare effects of the combination of dietary patterns and conventional lifestyle behaviors, such as alcohol drinking, tobacco smoking, and physical activity, in relation to general and central obesity. We tested the interaction between dietary patterns and lifestyle factors by creating categorical interactions terms and performed a likelihood ratio test to compare the difference of models with and without the interactions terms. All statistical analyses were performed with SAS version 9.3 (SAS Institute Inc., Cary, NC, USA). Significance was defined as *p* < 0.05.

**Table 1:** Dietary patterns identified by K-means cluster procedure ^1^.

	Dietary Patterns						Overall Mean (SD)
	Traditional Southern Dietary Pattern		Traditional Northern Dietary Pattern		Western/New Affluence Dietary Pattern		
Food group, day/week							
Rice	7.0	+ +	1.4	− − −	5.6	=	5.3 (2.6)
Wheat	1.7	− −	7.0	+ + +	5.0	+	3.7 (2.9)
Other staple foods	0.4	−	4.0	+ + +	1.2	−	1.4 (2.3)
Meat	3.9	=	1.4	−−	5.5	+ +	3.7 (2.5)
Poultry	0.8	=	0.1	− −	1.4	+ +	0.8 (1.0)
Fish	1.5	=	0.1	− −	2.3	+ +	1.4 (1.6)
Eggs	1.8	−	2.4	=	4.2	+ +	2.5 (2.2)
Fresh vegetables	6.9	=	6.6		7.0	+	6.8 (0.8)
Soybean	1.6	=	0.9	−	2.6	+ +	1.7 (1.6)
Preserved vegetables	2.3	=	1.5	−	2.5	+	2.2 (2.4)
Fresh fruit	1.9	− −	1.3	− −	5.3	+ + +	2.6 (2.5)
Dairy products	0.2	− −	0.4	−	3.2	+ + +	0.9 (2.1)
Beverage group, g/week							
Beer	1.2	=	0.9	=	14.4	+	4.1 (33.6)
Rice wine	5.9	=	<0.1	=	1.1	=	3.5 (35.3)
Wine	<0.1	=	<0.1	=	0.4	=	0.1 (3.6)
Heavy spirit (≥40%)	31.1	=	10.8	−	22.3	=	24.3 (113.0)
Light spirit (<40%)	13.9	=	6.1	=	4.4	=	9.9 (68.4)
Green tea	5.8	=	3.5	−	11.3	+	6.5 (15.9)
Oolong tea	0.5	=	<0.1	=	0.6	=	0.4 (4.5)
Black tea	1.8	+	<0.1	−	0.2	−	1.0 (7.6)
Other tea	<0.1	=	<0.1	=	<0.1	=	0.0 (0.7)

^1^ SD, standard deviation; The sign − indicates variation below the mean frequency of intake, while + indicates variation above the mean frequency of intake. For example, we used + (or −) for 0.1–0.49 SD units (SDUs); + + (or − −) for 0.5–0.99 SDUs; and + + + (or − − −) for 1.0–1.99 SDUs; The sign = means equal to mean frequency of intake; For example, for “rice”, the “traditional southern dietary pattern” had a mean intake of 7.0 days/week, which is 0.65 (*i.e.*, (7.0–5.3)/2.6) SDUs above the mean intake, so the corresponding sign (+ +) is used to indicate 0.5–0.99 SDUs above the mean.

## 3. Results

Three unique dietary patterns were identified in the present Chinese population (Table 1). The first cluster, the traditional southern dietary pattern, represented a typical traditional diet in South China, characterized by high intakes of rice but low intakes of wheat as staples. The second cluster was a traditional northern dietary pattern that, on the other hand, was characterized by high intakes of wheat and other staples, but low intakes of rice, meat, poultry, fish and fresh fruit. The third cluster, labeled as the Western/new affluence dietary pattern, was characterized by high consumption of fresh fruit and protein products such as meat, poultry, fish, eggs and dairy products.

Among the study population, 53.9% of the participants followed the traditional southern dietary pattern, 23.4% followed the traditional northern dietary pattern, and 22.7% followed the Western/new affluence dietary pattern. Participants who followed the Western/new affluence dietary pattern had better education and higher household income, were more likely to be a current drinker but less likely to be a current smoker, and were slightly less physically active (Table 2). Not surprisingly, those clustered into the traditional northern dietary pattern mainly lived in northern rural areas, while those with the traditional southern dietary patterns mainly lived in the southern rural areas.

**Table 2:** Selected characteristics of Chinese adults aged 30–79 according to dietary patterns ^1^.

	Traditional Southern Dietary Pattern	Traditional Northern Dietary Pattern	Western/New Affluence Dietary Pattern
*n* (%)	255,758 (53.9)	110,962 (23.4)	107,472 (22.7)
Female, %	59.3	59.2	58.4
Age, years	51.7 ± 0.02	49.8 ± 0.03	50.4 ± 0.03
Urban area, %	40.1	6.8	85.2
Southern area, %	94.2	0.7	43.6
Married, %	93.0	92.9	93.6
High school and above, %	38.3	38.0	83.6
Annual household income, %			
<10,000 Yuan RMB	24.2	56.5	8.3
10,000–19,999 Yuan RMB	27.7	33.2	27.6
≥20,000 Yuan RMB	48.1	10.3	64.1
Current drinker, %	8.1	3.2	10.3
Current smoker, %	13.3	11.4	8.8
Physical activity, Met-hour/day	22.7 ± 0.03	23.0 ± 0.04	18.9 ± 0.04

^1^ Values are age and sex adjusted percent or mean ± standard error (SE); All values in rows except sex are statistically different, *p* < 0.05; Met: metabolic equivalent task; Yuan RMB: unit of Chinese money.

**Figure 1:**
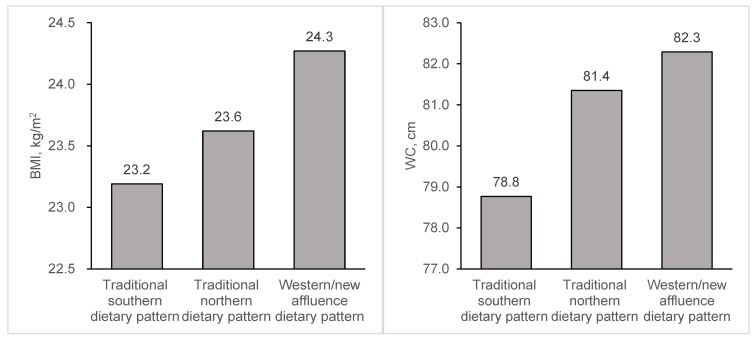
Age and sex adjusted means of body mass index (BMI) and waist circumference (WC) by dietary patterns in Chinese adults aged 30–79 years.

Age and sex adjusted means of anthropometric measures for three dietary patterns are presented in Figure 1. In general, the individuals who followed the traditional southern dietary pattern had the lowest BMI and WC, and those following a Western/new affluence dietary pattern had the highest BMI and WC (*p* values < 0.05). The differences remained statistically significant after adjustment for potential confounders, except in those following a traditional northern dietary pattern, who had the highest multivariable adjusted WC (Supplemental Table S4). The associations of dietary patterns with general and central obesity were similar (Table 3). Compared with the individuals following the traditional southern dietary pattern, those following the Western/new affluence dietary pattern were more likely to be generally obese (PR = 1.70; 95% CI = 1.67–1.74) and centrally obese (PR = 1.38; 95% CI = 1.37–1.40), and those following traditional northern dietary patterns were more likely to be generally obese (PR = 1.39; 95% CI = 1.36–1.42) and centrally obese (PR = 1.21; 95% CI = 1.20–1.22). The associations for both general and central obesity were attenuated after controlling for potential confounders but were still significant. Furthermore, such associations did not materially change when we further excluded deaths within two years after the baseline enrollment or the participants who tried to reduce weight during the last 12 months (data not shown).

We also assessed the joint effects of dietary patterns with conventional lifestyle behaviors (Table 4), the multivariable-adjusted associations of dietary patterns with general and central obesity were modified by alcohol drinking, tobacco smoking, and physical activity (all *p* values for interaction <0.05). A 28% and 24% excessive risk of general and central obesity respectively were observed among the current drinkers who had a Western/new affluence dietary pattern compared with a non-current drinker who had a traditional southern dietary pattern (*p* < 0.05). Furthermore, a 7% of elevated risk of central obesity was observed among current smokers who had a Western/new affluence dietary pattern (95% CI: 1.05–1.09); in spite of that, current smokers were generally associated with a significantly lower risk of general and central obesity compared with those without these lifestyle behaviors in each dietary pattern. Physical activity was negatively associated with general and central obesity in each dietary pattern (all *p* values for trend <0.05). Participants with a traditional northern dietary pattern combined with the most active (≥25.31 MET-hours/day), had the lowest risk of general obesity (PR = 0.59, 95% CI = 0.56–0.62), and those with the traditional southern dietary pattern in this group had the lowest risk of central obesity (PR = 0.82, 95% CI = 0.81–0.83).

**Table 3:** Multivariate adjusted prevalence ratios for general and central obesity by dietary patterns in Chinese adults aged 30–79 years ^1,2^.

	Traditional Southern Dietary Pattern	Traditional Northern Dietary Pattern	Western/New Affluence Dietary Pattern
General obesity			
No. of cases (%)	20,512 (8.02)	12,404 (11.18)	14,678 (13.66)
Crude	1.00	1.39 (1.36–1.42)	1.70 (1.67–1.74)
Model 1	1.00	1.41 (1.38–1.44)	1.71 (1.68–1.75)
Model 2	1.00	1.05 (1.01–1.09)	1.08 (1.05–1.10)
Model 3	1.00	1.05 (1.02–1.09)	1.06 (1.03–1.08)
Central obesity			
No. of cases (%)	90,783 (35.50)	47,694 (42.98)	52,813 (49.14)
Crude	1.00	1.21 (1.20–1.22)	1.38 (1.37–1.40)
Model 1	1.00	1.24 (1.23–1.25)	1.40 (1.39–1.41)
Model 2	1.00	1.17 (1.16–1.19)	1.08 (1.07–1.10)
Model 3	1.00	1.17 (1.15–1.18)	1.07 (1.06–1.08)

^1^ Values are prevalence ratios and 95% CIs unless specified. General obesity: BMI ≥ 28 kg/m^2^, Central obesity: WC ≥ 85 cm for men, ≥80 cm for women; ^2^ Crude: unadjusted model. Model 1: adjusted for age and sex. Model 2: model 1 + study area, marital status, education level, household income. Model 3: model 2 + alcohol consumption, smoking status, and physical activity.

**Table 4:** Joint effect of dietary patterns and lifestyle factors in relation to general obesity and central adiposity among Chinese adults aged 30–79 years ^1,2^.

	Traditional Southern Dietary Pattern	Traditional Northern Dietary Pattern	Western/New Affluence Dietary Pattern	*P* for Interaction
General obesity				
Current drinker				
No	1.00	1.04 (1.00–1.08)	1.03 (1.00–1.06)	<0.001
Yes	1.06 (1.02–1.10)	1.19 (1.12–1.28)	1.28 (1.23–1.34)	
Current smoker				
No	1.00	1.10 (1.06–1.14)	1.00 (0.97–1.03)	<0.001
Yes	0.69 (0.67–0.72)	0.56 (0.53–0.60)	0.92 (0.88–0.96)	
Physical activity				
T1	1.00	1.28 (1.23–1.33)	1.20 (1.16–1.24)	<0.001
T2	0.93 (0.90–0.96)	0.98 (0.93–1.03)	0.95 (0.91–0.98)	
T3	0.85 (0.82–0.87)	0.59 (0.56–0.62)	0.78 (0.74–0.81)	
Central adiposity				
Current drinker				
No	1.00	1.11 (1.10–1.13)	1.02 (1.01–1.03)	<0.001
Yes	1.03 (1.01–1.04)	1.19 (1.16–1.22)	1.24 (1.22–1.26)	
Current smoker				
No	1.00	1.19 (1.18–1.21)	1.00 (0.99–1.01)	<0.001
Yes	0.81 (0.80–0.82)	0.84 (0.82–0.86)	1.07 (1.05–1.09)	
Physical activity				
T1	1.00	1.16 (1.14–1.17)	1.10 (1.09–1.11)	<0.001
T2	0.92 (0.90–0.93)	1.11 (1.09–1.13)	0.95 (0.94–0.97)	
T3	0.82 (0.81–0.83)	0.98 (0.96–1.00)	0.87 (0.85–0.88)	

^1^ General obesity: Body mass index (BMI) ≥ 28 kg/m^2^; Central obesity: Waist circumference (WC) ≥ 85 cm for men, ≥80 cm for women; Physical activity was categorized into tertiles, T1: <12.29 metabolic equivalent task (MET-) hours/day, T2: 12.29–25.30 MET-hours/day, T3: ≥25.31 MET-hours/day; ^2^ Values are presented as prevalence ratios (95% CI) adjusted for age, sex, study area, marital status, education level, household income, alcohol consumption, smoking status, and physical activity.

## 4. Discussion

In the present study of a large sample of Chinese adults, we found that the traditional southern dietary pattern, characterized by high intakes of rice but low intakes of wheat as staple foods, was associated with the lowest risk of general and central obesity. Compared with the traditional southern dietary pattern, the traditional northern dietary pattern was characterized by high intakes of wheat as a staple food, but low intakes of rice, meat, poultry, fish, and fresh fruit, was associated with an elevated risk of general and central obesity. The Western/new affluence dietary pattern, characterized by high consumption of fresh fruit and protein products such as red meat, poultry, fish, eggs and dairy products, was also associated with an increased risk of general and central obesity. These associations were independent of the socio-demographic and conventional lifestyle behaviors.

The dietary patterns in this present study were constructed using cluster analysis combined with factor analysis, which derived more reproducible and interpretable results. The three dietary patterns fully captured the geographical, socioeconomic and dietary characteristics in the Chinese population, and were consistent with previously reported results from a large-scale, nationally representative sample of Chinese adults with a validated semi-quantitative food frequency questionnaire [13,14,15]. Similar dietary patterns were also reported from three consecutive 24-hours recalls of dietary intake among young Chinese women aged 18–44 years [16].

The risk differences between the traditional southern dietary pattern and traditional northern dietary pattern in terms of general and central obesity were reported in previous cross-sectional studies [14,15,16,26]. In a five-year prospective study conducted in Jiangsu, Shi *et al.* [27] reported that adoption of a traditional southern dietary pattern was associated with less weight gain. These two traditional Chinese dietary patterns, which mainly differ in taking rice or wheat as a staple, had remarkably different distributions in northern and southern China. Previous prevalence studies on obesity also revealed this geographic difference [28,29]. One study that used the percentage of rice in the staple food as an index of a staple food pattern, reported that the index was inversely related to weight gain [30]. The underlying mechanism is unclear; it may have some connection with lipid alteration caused by carbohydrate intake [31,32] and insufficiency of micronutrients [33] in the traditional northern dietary pattern.

The Western/new affluence dietary pattern was suggested to be associated with an elevated risk of general and central obesity. This is consistent with a body of literature conducted in different countries and ethnicities [14,15,34,35,36,37,38,39]. Meat consumption, one of the key features of the Western/new affluence dietary pattern, was reported to be strongly associated with weight gain in several prospective studies [3,40,41]. The mechanism underlying the positive association is unclear: several hypotheses have been made regarding energy density in appetite control and an underlying detrimental lifestyle beyond the dietary patterns [40]. However, in the present study, which took into account conventional behavioral factors as potential confounders in this relationship, the associations were only slightly attenuated.

Previous studies reported that lifestyle behaviors, such as alcohol drinking [42,43,44] and tobacco smoking [45,46], were associated with BMI or measures of obesity. These associations were observed in the present study. In addition, we found that the associations of dietary patterns in relation to general and central obesity were modified by these lifestyle factors. Current drinkers and current smokers who have a Western/new affluence dietary pattern have the highest risk of general and central obesity. Physical activity as a form of energy expenditure, is often regarded as a standard clinical recommendation for generally or centrally obese individuals [47]. Previous studies reported regular physical activity had a protective effect against long-term gain in weight [3,48,49], BMI [50], or central obesity [15]. This protective effect was stronger among individuals who followed a traditional northern dietary pattern for general obesity, and those who followed a Western/new affluence dietary pattern for central obesity according the joint analysis. The energetics of diet composition may explain this difference. The carbohydrate from wheat and other staple food contributes major dietary energy intake in the traditional northern dietary pattern, while protein-rich diet components, such as meat, poultry, fish, egg, and dairy products, provide most of dietary energy in the Western/new affluence dietary pattern. Evidence has shown that high protein diets produced greater improvements than high carbohydrate diets in both short and long term weight maintenance. The former was shown to be associated with an increased ratio of fat/muscle loss and positive changes of blood lipids profile, such as lowing triacylglycerols (TAG) and ratio of TAG/high density lipoprotein cholesterol (HDL-C) [51,52]. Similar to these findings, we found that individuals who followed a Western/new affluence dietary pattern with higher physical activity, were associated a lower risk of central obesity as comparing to those followed a traditional northern dietary pattern with the same physical activity level. Proposed mechanisms include increased hunger, lower satiety, or great calorie intake after consuming carbohydrates *vs.* a protein-rich diet [53]. This information could be helpful when it was translated into diet recommendations in weight control programs.

The present study is thus far the largest study of the dietary patterns on general and central obesity with objective anthropometric measurement in the Chinese population. However, some points should be considered in interpreting our findings. First, the nature of cross-sectional design limits causal inference. However, the observed associations remained after a further exclusion of participants who tried to reduce weight by dieting and using weight-loss drugs during the last 12 months and those who had chronic conditions at baseline survey. Secondly, the limitation of our questionnaires should be considered. We only assessed 12 crude food groups that are common in the Chinese diet; other food groups that may be related to general and central obesity were not collected, such as sweets, other than non-alcoholic beverages. Although the major dietary patterns in Chinese adults were well captured in our study, only the frequencies but not the quantity of the major food groups were collected. Thus, it was difficult to calculate and adjust for total energy intake. However, according to a previous study in Iranian women, additional adjustment of energy intake could not affect the magnitude and significance of BMI and WC, or use general and central obesity as an outcome [39]. In addition, shifting the quantitative focus on food calories to qualitative focus on consuming foods of different types, could address different food-induced physiological pathways and different metabolic effects [54]. Thirdly, although we excluded the participants who had chronic conditions at the baseline and adjusted for a wide range of socio-demographic and behavioral variables in present analysis, the possibility of residual confounding, such as ethnicity, could not be excluded. We didn’t collect detailed dietary information on food processing, which limited our ability to comprehensively adjust for other specific dietary factors. Furthermore, we constructed the major three dietary patterns, which captured the most typical diet habits in Chinese population. However, some minor dietary patterns might also be associated with general and central obesity.

## 5. Conclusions

In conclusion, we found that the traditional southern dietary pattern was related to a lower risk of general and central obesity independent of socio-demographic and lifestyle behavior factors, while the Western/new affluence dietary pattern was associated with increased risk of these conditions. Although the differences in physical measures and the risk of general and central obesity were very moderate, a slight difference in the population level may have important public health significance. Hence, the results from the present study could be integrated into community-based health promotion and intervention programs of chronic diseases. Furthermore, future studies are required to elucidate the mechanisms beyond the well-known risk factors of obesity.

## Supplementary Files

Supplementary File 1

## China Kadoorie Biobank Collaborative Group

**International Steering Committee:** Junshi Chen, Zhengming Chen, Rory Collins, Liming Li (PI), Richard Peto. **International Co-ordinating Centre, Oxford:** Derrick Bennett, Yumei Chang, Yiping Chen, Zhengming Chen, Robert Clarke, Huaidong Du, Xuejuan Fan, Haiyan Gao, Simon Gilbert, Andri Iona, Rene Kerosi, Ling Kong, Om Kurmi, Garry Lancaster, John McDonnell, Winnie Mei, Iona Millwood, Qunhua Nie, Jayakrishnan Radhakrishnan, Paul Ryder, Sam Sansome, Dan Schmidt, Paul Sherliker, Margaret Smith, Rajani Sohoni, Robin Walters, Jenny Wang, Lin Wang, Alex Williams, Ling Yang, Xiaoming Yang. **National Coordinating Centre, Beijing:** Zheng Bian, Ge Chen, Lei Guo, Yu Guo, Bingyang Han, Can Hou, Peng Liu, Jun Lv, Pei Pei, Shuzhen Qu, Yunlong Tan, Canqing Yu, Huiyan Zhou. **10 Regional Coordinating Centres:** **Qingdao** Qingdao CDC: Zengchang Pang, Shaojie Wang, Yun Zhang, Kui Zhang. Licang CDC: Silu Liu, Wei Hou. **Heilongjiang** Provincial CDC: Zhonghou Zhao, Shumei Liu, Zhigang Pang. Nangang CDC: Weijia Feng, Shuling Wu, Liqiu Yang, Huili Han, Hui He, Bo Yu. **Hainan** Provincial CDC: Xianhai Pan, Shanqing Wang, Hongmei Wang. Meilan CDC: Xinhua Hao, Chunxing Chen, Shuxiong Lin, Xiangyang Zheng. **Jiangsu** Provincial CDC: Xiaoshu Hu, Minghao Zhou, Ming Wu, Ran Tao. Suzhou CDC: Yeyuan Wang, Yihe Hu, Liangcai Ma, Renxian Zhou, Guanqun Xu, Yan Lu. **Guangxi** Provincial CDC: Baiqing Dong, Naying Chen, Ying Huang. Liuzhou CDC: Mingqiang Li, Jinhuai Meng, Zhigao Gan, Jiujiu Xu, Yun Liu, Jingxin Qing. **Sichuan** Provincial CDC: Xianping Wu, Yali Gao, Ningmei Zhang. Pengzhou CDC: Guojin Luo, Xiangsan Que, Xiaofang Chen. **Gansu** Provincial CDC: Pengfei Ge, Jian He, Xiaolan Ren. Maiji CDC: Hui Zhang, Enke Mao, Guanzhong Li, Zhongxiao Li, Jun He, Yulong Lei, Xiaoping Wang. **Henan** Provincial CDC: Guohua Liu, Baoyu Zhu, Gang Zhou, Shixian Feng. Huixian CDC: Yulian Gao, Tianyou He, Li Jiang, Jianhua Qin, Huarong Sun. **Zhejiang** Provincial CDC: Liqun Liu, Min Yu, Yaping Chen, Ruying Hu. Tongxiang CDC: Zhixiang Hu, Jianjin Hu, Yijian Qian, Zhiying Wu, Chunmei Wang, Lingli Chen. **Hunan** Provincial CDC: Wen Liu, Guangchun Li, Huilin Liu. Liuyang CDC: Xiangquan Long, Xin Xu, Youping Xiong, Zhongwen Tan, Xuqiu Xie, Yunfang Peng, Weifang Jia.
