# Supplementary material for: Major Dietary Patterns in Relation to General and Central Obesity among Chinese Adults
Source: Nutrients. 2015 Jul 15;7(7):5834–49. doi: 10.3390/nu7075253 (PMC4517030; doi:10.3390/nu7075253)
Supplement: Supplementary File 1 [file nutrients-07-05253-s001.docx]

Supplemental Materials


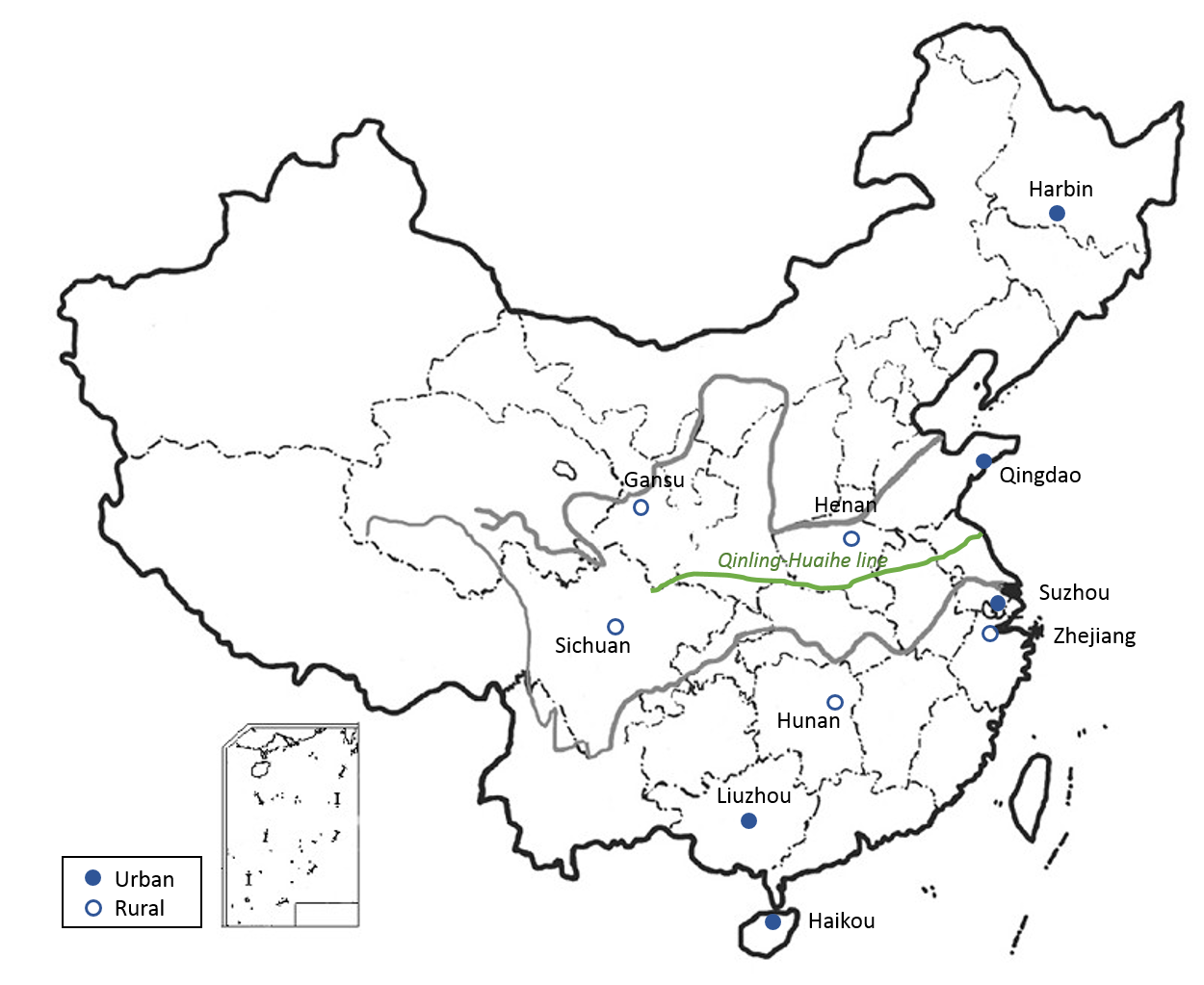


**Figure S1.** Locations of the 10 study areas of the China Kadoorie Biobank. Solid circles indicate urban areas and open circles indicate rural areas. Circles above the green line
(Qinling-Huaihe line) indicate northern areas and those below the line indicate southern areas.

**Table S1.** Correlation coefficient comparing food intake at baseline with a repeat questionnaire within 1 year (*n* = 926).

|  | **Spearman Correlation Coefficient** | | **Polychoric Correlation Coefficient ^1^** |
| --- | --- | --- | --- |
|  | **Crude** | **Age-, Sex-Adjusted** |  |
| Food group |  |  |  |
| Rice | 0.75 | 0.74 | 0.83 |
| Wheat | 0.76 | 0.76 | 0.82 |
| Other staple foods | 0.45 | 0.44 | 0.54 |
| Meat | 0.48 | 0.48 | 0.60 |
| Poultry | 0.48 | 0.48 | 0.56 |
| Fish | 0.60 | 0.60 | 0.69 |
| Eggs | 0.58 | 0.58 | 0.66 |
| Fresh vegetables | 0.17 | 0.16 | 0.52 |
| Soybean | 0.35 | 0.35 | 0.42 |
| Preserved vegetables | 0.54 | 0.54 | 0.60 |
| Fresh fruit | 0.55 | 0.54 | 0.61 |
| Dairy products | 0.66 | 0.66 | 0.78 |
| Beverage group |  |  |  |
| Beer | 0.31 | 0.27 |  |
| Rice wine | 0.43 | 0.42 |  |
| Wine | 0.40 | 0.40 |  |
| Heavy spirit (≥40%) | 0.64 | 0.60 |  |
| Light spirit (<40%) | 0.40 | 0.38 |  |
| Green tea | 0.71 | 0.69 |  |
| Oolong tea | 0.72 | 0.71 |  |
| Black tea | 0.63 | 0.63 |  |
| Other tea | –^2^ | –^2^ |  |

^1^ Only food groups were calculated the polychoric correlation coefficient, and their frequencies were regarded as ordinal variables; ^2^ The sign – means the data are not applicable to calculate the Spearman correlation coefficient because they are singular and kappa = 1.

**Table S2.** Factor loading matrix of two major factor solutions after varimax rotation ^1^.

| **Food or Beverage Group** | **Factor 1** | **Factor 2** |
| --- | --- | --- |
| Rice | **−0.836** | 0.208 |
| Wheat | **0.845** | 0.116 |
| Other staple | **0.703** | −0.164 |
| Meat | −0.361 | **0.600** |
| Poultry | −0.347 | **0.558** |
| Fish | −0.347 | **0.525** |
| Eggs | 0.325 | **0.509** |
| Fresh vegetables | −0.064 | 0.200 |
| Soybean | −0.124 | **0.471** |
| Preserved vegetables | −0.151 | 0.128 |
| Fresh fruit | 0.033 | **0.698** |
| Dairy | 0.234 | **0.633** |
| Beer | 0.060 | 0.198 |
| Rice wine | −0.142 | 0.002 |
| Wine | <0.001 | 0.059 |
| Heavy spirit (≥40%) | −0.078 | −0.002 |
| Light spirit (<40%) | −0.111 | −0.016 |
| Green tea | −0.005 | 0.225 |
| Oolong tea | −0.075 | 0.069 |
| Black tea | −0.189 | −0.042 |
| Other tea | −0.011 | 0.006 |
| Eigen Value | 3.08 | 2.04 |
| Percentage of variances (%) explained | 14.68 | 9.70 |

^1^ Extraction method: principal component analysis with varimax rotation. Figures in bold indicate absolute factor loading are more than cut-off score of 0.40 and considered to belong to the corresponding dimension in the column.

**Table S3.** Classification of subjects by cluster analysis using factor score ^1^

|  | **Cluster 1:** | **Cluster 2:** | **Cluster 3:** |
| --- | --- | --- | --- |
|  | **Traditional Southern Dietary Pattern** | **Traditional Northern Dietary Pattern** | **Western/New Affluence Dietary Pattern** |
| Factor 1 | −0.74 ± 0.37 | 1.38 ± 0.42 | 0.32 ± 0.72 |
| Factor 2 | −0.28 ± 0.58 | −0.75 ± 0.53 | 1.45 ± 0.68 |

^1^ Data are shown as mean ± standard deviation (SD). All *p* are <0.001 by analysis of variance. The values in the same row were significantly different by Turkey-Kramer adjustment for multiple comparisons at *p* < 0.001.

**Table S4.** Multivariate adjusted means for anthropometric measures by dietary patterns in Chinese adults aged 30–79 years ^1,2^.

|  | **Traditional Southern Dietary Pattern** | **Traditional Northern Dietary Pattern** | **Western/New Affluence Dietary Pattern** |
| --- | --- | --- | --- |
| BMI, kg/m^2^ |  |  |  |
| Crude | 23.2 (23.2–23.2) | 23.7 (23.6–23.7) | 24.3 (24.3–24.3) |
| Model 1 | 23.2 (23.2–23.2) | 23.6 (23.6–23.6) | 24.3 (24.3–24.3) |
| Model 2 | 23.4 (23.4–23.4) | 23.4 (23.3–23.4) | 23.6 (23.5–23.6) |
| Model 3 | 23.6 (23.6–23.7) | 23.7 (23.6–23.7) | 23.8 (23.8–23.8) |
| WC, cm |  |  |  |
| Crude | 78.6 (78.5–78.6) | 81.0 (80.9–81.0) | 82.0 (81.9–82.1) |
| Model 1 | 78.8 (78.7–78.8) | 81.4 (81.3–81.4) | 82.3 (82.2–82.3) |
| Model 2 | 79.5 (79.4–79.6) | 80.9 (80.8–81.0) | 80.4 (80.3–80.4) |
| Model 3 | 80.2 (80.1–80.3) | 81.8 (81.7–81.9) | 80.9 (80.9–81.0) |

^1^ BMI: body mass index; WC: waist circumference; ^2^ Crude: unadjusted model. Model 1: adjusted for
age and sex; Model 2: model 1 + study area, marital status, education level, household income;
Model 3: model 2 + alcohol consumption, smoking status, and physical activity.
